# Real-world success of de-escalating upadacitinib induction dosing in ulcerative colitis is predicted by steroid-free clinical and biochemical remission, not induction duration

**DOI:** 10.1093/crocol/otag111

**Published:** 2026-10-29

**Authors:** Raj M Dalal, Sarah Rotondo-Trivette, Alec R Mason, Lynn Leveille, Jianyi Yin, Chelsea Missi, Moheb Boktor, David I Fudman

**Affiliations:** Department of Internal Medicine, University of Texas Southwestern Medical Center, Dallas, TX 75390, United States; Division of Digestive and Liver Diseases, University of Texas Southwestern Medical Center, Dallas, TX 75390, United States; Department of Internal Medicine, University of Texas Southwestern Medical Center, Dallas, TX 75390, United States; Department of Internal Medicine, University of Texas Southwestern Medical Center, Dallas, TX 75390, United States; Division of Digestive and Liver Diseases, University of Texas Southwestern Medical Center, Dallas, TX 75390, United States; Division of Digestive and Liver Diseases, University of Texas Southwestern Medical Center, Dallas, TX 75390, United States; Division of Digestive and Liver Diseases, University of Texas Southwestern Medical Center, Dallas, TX 75390, United States; Division of Digestive and Liver Diseases, University of Texas Southwestern Medical Center, Dallas, TX 75390, United States

**Keywords:** Janus Kinase inhibitors, upadacitinib, fecal calprotectin, biomarkers, ulcerative colitis, inflammatory bowel disease

## Abstract

**Background:**

Data guiding upadacitinib dose de-escalation in ulcerative colitis (UC) are limited. We evaluated factors associated with UC-related complications (UCRC) after upadacitinib was reduced from 45 mg daily.

**Methods:**

This retrospective study included adults with UC at a tertiary center who underwent upadacitinib de-escalation (4/2022-11/2025). The primary outcome was a 12-month composite of UCRC (upadacitinib re-escalation or discontinuation, new systemic steroids, new advanced therapy, hospitalization, or colectomy). Predictors of UCRC after de-escalation were assessed using Cox proportional hazards modeling, including steroid-free clinical remission (SFCR), defined as a stool frequency subscore of 0 or 1 and a rectal bleeding subscore of 0 without systemic steroid use, and biochemical remission, defined as a fecal calprotectin (FC) < 150 μg/g or a C-reactive protein (CRP) < 5 mg/L when FC was unavailable. Optimal CRP and FC cut points were identified via ROC curves.

**Results:**

Among 70 patients, 25 (35.7%) experienced UCRC (median follow-up 56 weeks). Induction duration, prior advanced therapy exposure, and endoscopic severity did not differ significantly between those with and without UCRC. Patients without UCRC were more likely to have achieved SFCR (51.1% vs 20.0%, *P*=.01) and biochemical remission (53.3% vs 28.0%, *P*=.04) prior to de-escalation, with lower median CRP (0.8 vs 1.5 mg/L, *P*=.04) and FC (79.5 vs 208.0 μg/g, *P*=.03). In multivariable analysis, achieving SFCR (aHR 0.33, 95% CI, 0.12-0.91) and biochemical remission (aHR 0.34, 95% CI, 0.13-0.85) independently predicted lower UCRC risk, and achieving both conferred additional UCAE-free survival benefit. Optimal cut points predicting UCRC were CRP > 12.7 mg/L (sensitivity 40%, specificity 93%) and FC > 58.7 μg/g (sensitivity 100%, specificity 47%).

**Conclusions:**

SFCR and biochemical remission prior to upadacitinib de-escalation, but not induction duration, predict lower UCRC risk. Upadacitinib de-escalation should be guided by response assessment rather than a predetermined induction duration.

## Introduction

Janus kinase (JAK) inhibitors are oral small molecules increasingly used to treat a variety of immune-mediated inflammatory diseases, including inflammatory bowel disease (IBD).^1^ Both the efficacy and safety of this drug class for the treatment of IBD are dose-dependent.^2–6^ Upadacitinib is the second JAK inhibitor approved for ulcerative colitis (UC), designed with preferential JAK1 selectivity in an effort to optimize its efficacy and safety over less selective JAK inhibition.^7^^,^^8^ In a phase 2b dose-ranging study for UC, the highest tested dose of 45 mg demonstrated the highest efficacy.^9^ This dose was selected for induction in the phase 3 program, followed by maintenance with either 15 mg or 30 mg daily.^10^

Based on these trials, the FDA approved upadacitinib for the treatment of moderately-to-severely active UC at a dose of 45 mg daily for an 8-week induction phase followed by de-escalation to the lowest effective maintenance dose of either 15 mg or 30 mg.^11^ However, clinical response to upadacitinib is not universally achieved after 8 weeks of induction, and extended induction is required in certain patients. Among non-responders who continued open-label upadacitinib 45 mg daily in the phase 3 program, 59% achieved clinical response after an additional 8 weeks of induction dosing.^12^

Additionally, disease relapse following dose de-escalation remains a significant concern. Clinical trial and real-world data demonstrate that among patients who achieve clinical response with induction, a significant proportion fail to achieve clinical remission on maintenance dosing of 15 mg or 30 mg, in a dose-dependent manner.^13–15^ Moreover, not all patients will recapture clinical response upon re-escalation to 45 mg daily.^14^^,^^16^

Consequently, when and how to de-escalate upadacitinib in real-world practice remains unclear. Current FDA safety labeling for both JAK inhibitors approved to treat UC (tofacitinib and upadacitinib) mandates a step-down from high-dose induction to lower-dose maintenance regimens to the lowest effective dose. For tofacitinib, the RIVETING trial—which randomized patients with UC in stable remission on 10 mg twice daily to de-escalation to 5 mg twice daily or no dose change—demonstrated that endoscopic remission (Mayo endoscopic subscore 0 vs 1) and no history of tumor necrosis factor inhibitor (TNFi) failure were associated with sustained remission after dose reduction.^17^ However, comparable data to guide dose de-escalation in upadacitinib remain scarce, underscoring the importance of identifying factors that may predict the success of dose reduction.

Moreover, multiple lines of evidence have demonstrated that fecal calprotectin (FC) can predict the relapse of IBD following the therapeutic de-escalation of TNF inhibitors,^18–20^ but objective biochemical markers have not been formally evaluated in the context of upadacitinib dose de-escalation.

Therefore, we sought to identify factors associated with an increased risk of UC-related complications (UCRC) following upadacitinib dose de-escalation.

## Materials and methods

### Study population

We conducted a retrospective cohort study of patients treated with upadacitinib for UC at a tertiary academic medical center. Patients were included if they had a diagnosis of UC that was documented by a gastroenterologist and were treated with upadacitinib 45 mg for at least 8 weeks between 4/2022 and 11/2025. We excluded patients younger than 18 years old and those who lacked follow up data for at least 8 weeks after upadacitinib initiation. Additionally, we excluded patients treated with upadacitinib only as a planned bridge to monotherapy with another advanced therapy,^21^ patients with a diagnosis of indeterminate colitis or Crohn’s disease, and patients with a stoma or ileal pouch anal anastomosis. Charts were initially screened by diagnosis code and prescription history, then inclusion criteria were confirmed by manual review.

### Definition of co-variates and outcomes

Patient demographic and clinical data were collected via chart review. Demographic data included sex, race, ethnicity, BMI, and age at the time of upadacitinib initiation. Baseline clinical variables prior to upadacitinib initiation included disease duration, disease distribution (categorized as proctitis, left-sided, or extensive), prior conventional or advanced therapy use, concurrent use of systemic steroids, concurrent use of a second advanced therapy, and hospitalization at the time of upadacitinib initiation.

To assess baseline disease severity, we recorded clinical disease activity prior to initiation using the Patient-Reported Outcome 2 (PRO2) score, consisting of stool frequency score (SFS) and rectal bleeding score (RBS).^22^ Clinical remission was defined as SFS of 0 or 1 and RBS of 0. Moderate to severe clinical disease activity were defined as SFS of 2 or 3 and RBS of 2 or 3. All other combinations were classified as mild to moderate disease activity.

Additional measures of baseline disease activity included C-reactive protein (CRP), FC, and Mayo endoscopic subscores at the most recent time point within the 6 months prior to upadacitinib initiation.

### Assessment prior to dose de-escalation

Systemic steroid use, clinical disease activity, and biochemical markers were assessed using clinical data collected between 4 weeks after upadacitinib induction and the date of dose de-escalation. For patients with multiple results in this window, the assessment closest to the de-escalation data was analyzed. Steroid-free clinical remission (SFCR) prior to dose de-escalation was defined as SFS of 0 or 1 and RBS of 0 while off systemic steroids, and biochemical remission was defined as FC of less than 150 μg/g or a CRP less than 5 mg/L if FC was not available. We also recorded duration of induction and whether patients de-escalated to 30 mg or 15 mg.

### Study outcomes and safety monitoring

The primary outcome was a composite of UC-related complications (UCRC) within 12 months of upadacitinib de-escalation. This composite included: 1) UC-related initiation of systemic steroids; 2) upadacitinib dose re-escalation to 45 mg; 3) addition of another advanced therapy; 4) upadacitinib discontinuation following de-escalation; 5) UC-related hospitalization; 6) colectomy.

Upadacitinib-related adverse events were also manually extracted from review of clinical encounters. Major adverse events that were screened for included major adverse cardiovascular events (MACE), venous thromboembolism (VTE), herpes zoster reactivation, gastrointestinal perforation, and severe bleeding. Minor adverse events that were screened for included acne, weight gain, arthralgias, abdominal pain, and non-zoster infections. The association between duration of upadacitinib induction and the occurrence of upadacitinib-related adverse events was assessed using Chi square analysis.

### Statistical analysis

Baseline demographic and clinical variables were compared between patients who did and did not experience a UCRC within 12 months of de-escalation using Chi square for categorical variables and the Wilcoxon rank-sum test for continuous variables. To identify covariates that were associated with hazard of UCRC, a multivariate Cox proportional hazards model was created including variables with *P*-values of <.1 in univariate Cox proportional hazards analysis. In post-hoc analysis, receiver operating characteristic (ROC) curves were generated for FC and CRP, depicting their sensitivity and specificity for UCRC within 12 months. Youden’s J-statistics were then used to identify optimal cutoffs for prediction of UCRC. In addition, a univariable analysis was conducted to identify factors associated with prolongation of induction dosing. Statistical analysis was conducted in StataNow BE 19 (College Station, TX).

#### Ethical considerations

Data were anonymized prior to analysis to ensure confidentiality, in accordance with a study protocol approved by the local Institutional Review Board.

## Results

### Baseline patient characteristics

We identified 70 patients who met inclusion criteria (Table 1). Slightly over half (54.3%) of patients were male and 80% were white. The median age was 37.5 years (IQR 31-49). Most patients (78.6%) had extensive disease, and the median number of advanced therapies used prior to upadacitinib initiation was 3 (IQR 2-4), including prior infliximab exposure in 70%. Forty percent of patients were using systemic steroids at the time of upadacitinib initiation.

**Table 1:** Study population clinical and demographic characteristics.

	Total	No UCRC	UCRC	*P*
**TOTAL**	**70**	**45**	**25**	—
**Gender**				
** Male**	38 (54.3%)	21 (46.7%)	17 (68.0%)	.086
** Female**	32 (45.7%)	24 (53.3%)	8 (32.0%)	
**Race**				
** White**	56 (80.0%)	39 (86.7%)	17 (68.0%)	**.048**
** Black**	6 (8.6%)	4 (8.9%)	2 (8.0%)	
** Other/Unknown**	8 (11.4%)	2 (4.4%)	6 (24.0%)	
**Hispanic**	12 (17.1%)	9 (20.0%)	3 (12.0%)	.395
**Body mass index (kg/m^2^)**	26.3 [22.7, 30.1]	26.3 [22.1, 30.0]	26.7 [24.3, 30.3]	.323
**Age at induction (years)**	37.5 [31, 49]	39 [32, 54]	35 [29, 46]	.169
**Disease duration (years)**	9 [5, 16]	10 [6, 15.5]	9 [4, 16]	.694
**Disease distribution**				
** Proctitis**	1 (1.4%)	1 (2.2%)	0 (0.0%)	.603
** Left-sided**	14 (20.0%)	10 (22.2%)	4 (16.0%)	
** Extensive**	55 (78.6%)	34 (75.6%)	21 (84.0%)	
**Prior advanced therapies**	3 [2, 4]	3 [2, 4]	3 [2, 3]	.687
**Prior infliximab exposure**	49 (70.0%)	33 (73.3%)	16 (64.0%)	.414
**Hospitalized at induction**	10 (14.3%)	7 (15.6%)	3 (12.0%)	.684
**Systemic steroids at induction**	28 (40.0%)	17 (37.8%)	11 (44.0%)	.611
**Dual advanced therapy at induction**	10 (14.3%)	8 (17.8%)	2 (8.0%)	.263
**Pre-induction clinical disease activity**				
** Remission**	16 (22.9%)	12 (26.7%)	4 (16.0%)	.300
** Mild-to-moderate**	2 (2.9%)	2 (4.4%)	0 (0.0%)	
** Moderate-to-severe**	52 (74.3%)	31 (68.9%)	21 (84.0%)	
**Pre-induction biochemical markers**				
** C-reactive protein (mg/L)**	10.4 [3.0, 29.5]	5.8 [1.8. 21.9]	21 [10.3, 38.8]	**.015**
** Fecal calprotectin (**μ**g/g)**	978.5 [416.8, 2300.5]	916.4 [357.0, 1951.3]	1381.8 [751, 2557.4]	.080
**Pre-induction biochemical remission**	8 (11.4%)	7 (15.6%)	1 (4.0%)	.145
**Pre-induction mayo endoscopic subscore**				
** Mayo 0-1**	4 (5.7%)	3 (6.7%)	1 (4.0%)	.719
** Mayo 2**	17 (24.3%)	11 (24.4%)	6 (24.0%)	
** Mayo 3**	25 (35.7%)	19 (42.2%)	6 (24.0%)	
**Induction duration**				
** 8 Weeks**	36 (51.4%)	23 (51.1%)	13 (52.0%)	.240
** 9-16 Weeks**	16 (22.9%)	8 (17.8%)	8 (32.0%)	
** >16 Weeks**	18 (25.7%)	14 (31.1%)	4 (16.0%)	
**De-escalation dose**				
** 15 mg**	3 (4.3%)	1 (2.2%)	2 (8.0%)	.253
** 30 mg**	67 (95.7%)	44 (97.8%)	23 (92.0%)	
**Clinical disease activity at de-escalation**				
** Remission**	31 (44.3%)	24 (53.3%)	7 (28.0%)	.096
** Mild-to-moderate**	6 (8.6%)	4 (8.9%)	2 (8.0%)	
** Moderate-to-severe**	33 (47.1%)	17 (37.8%)	16 (64.0%)	
**SFCR prior to de-escalation**	28 (40.0%)	23 (51.1%)	5 (20.0%)	**.011**
**Biochemical markers prior to de-escalation**				
** C-reactive protein (mg/L)**	1.5 [0.2, 7.4]	0.8 [0.2, 5.7]	1.5 [0.6, 20.2]	**.041**
** Fecal calprotectin (**μ**g/g)**	116.1 [37.0, 357.0]	79.5 [15.0, 329.0]	208.0 [93.0, 464.9]	**.034**
**Biochemical remission prior to de-escalation**	31 (44.3%)	24 (53.3%)	7 (28.0%)	**.041**
**Days of follow-up after de-escalation**	394 [158, 690]	338 [139, 614]	285 [150, 548]	.849

Clinical and demographic baseline data for patients who underwent upadacitinib de-escalation. Results are reported as either number (percent) or median [IQR] for categorical and continuous variables, respectively. *P*-values shown are calculated via Chi-square for categorical variables and Wilcoxon rank-sum tests for continuous variables, comparing those with and without UC-related Complications. Bold values indicate statistical significance (P < .05).

UCRC, UC-related complications; SFCR, steroid-free clinical remission.

Moderate-to-severe clinical disease activity was present in 74.3% of patients prior to upadacitinib initiation. The median CRP and FC were 10.4 mg/L (IQR 3.0-29.5) and 978.5 μg/g (IQR 416.8-2300.5), respectively. Of the 46 patients that had an endoscopic assessment within 6 months prior to upadacitinib initiation, 42 (91.3%) had a Mayo endoscopic subscore of 2 or greater.

The duration of upadacitinib induction with 45 mg daily was 8 weeks for 51.4% of patients, 9-16 weeks for 22.9% of patients, and 17 or more weeks for 25.7% of patients (Table S1). Induction duration was significantly associated with pre-induction Mayo endoscopic sub-score and with FC measured at week 4-8 after induction. There was no significant association between induction duration and sex, race, BMI, age, or use of prior advanced therapy. Upon dose de-escalation, nearly all patients (95.7%) utilized 30 mg daily (Table 1).

### Primary outcome—UC complications

Of 70 patients, 25 (35.7%) experienced the composite endpoint of UCRC (Table 2). Among these, 2 patients underwent colectomy, 4 discontinued upadacitinib, 13 re-escalated upadacitinib to 45 mg, 3 utilized systemic steroids, and 3 had a second advanced therapy added to their regimen.

**Table 2:** UC-related complications.

	Total
**Any UCRC**	25 (35.7%)
**Colectomy**	2 (2.9%)
**Non-colectomy hospitalization**	0 (0.0%)
**Upadacitinib discontinuation**	4 (5.7%)
**Upadacitinib re-escalation**	13 (18.6%)
**Systemic corticosteroids**	3 (4.3%)
**Second advanced therapy**	3 (4.3%)

Frequency of the primary outcome, any UCRC, and specific UCRCs in this study, displayed as number and percentage of total study cohort.

### Factors associated with UCRC

In univariable analysis, there were no significant differences in induction duration, previous advanced therapy exposure, disease distribution, or pre-induction Mayo endoscopic score between those with and without UCRCs (Table 1). Patients with UCRC had higher CRP prior to upadacitinib induction (21 vs 5.8 mg/L, *P*=.015) and were more likely to be non-white (32% vs. 13.3%, *P*=.048).

Patients without UCRC were more likely to be in SFCR and biochemical remission prior to upadacitinib de-escalation (51.1% vs 20.0%, *P*=.011; 53.3% vs 28.0%, *P*=.041, respectively) and had lower median CRP and FC prior to de-escalation (0.8 vs 1.5 mg/L, *P*=.041; 79.5 vs 208.0 μg/g, *P*=.034, respectively). There was no difference in length of follow-up after de-escalation between those with and without UCRC (median 338 vs. 285 days, *P*=.849).

### Multivariate analysis and proposed biochemical cutoffs

Variables with *P* < .1 in univariable Cox proportional hazards analysis (Table S2) were selected for inclusion in multivariable analysis. In this multivariate Cox model examining factors associated with the hazard of UCRC that included clinical remission, biochemical remission, and race as covariates, there was a significantly lower hazard of UCRC in those that achieved SFCR prior to de-escalation (aHR 0.33, 95% CI, 0.12-0.91), in those that achieved biochemical remission prior to de-escalation (aHR 0.34, 95% CI, 0.13-0.85) and in white patients (aHR 0.20, 95% CI, 0.08-0.51) (Table 3).

**Table 3:** Multivariable cox proportional hazards model of UCRC hazard.

Covariate	aHR	95% CI	***P*-value**
**Steroid-free clinical remission**	0.334	[0.123, 0.906]	**.031**
**Biochemical remission**	0.336	[0.133, 0.850]	**.021**
**White (vs. non-white)**	0.202	[0.080, 0.512]	**.001**

Multivariable Cox proportional hazards model showing modeled adjusted hazard ratios (aHR) for SFCR, biochemical remission, and race. These variables were selected for inclusion in multivariable modeling based on having *P* < .1 in univariate Cox modeling. Bold values indicate statistical significance (P < .05).

Patients who achieved neither SFCR nor biochemical remission had a modeled UCRC rate of approximately 70% (Figure 1). Patients achieving SFCR alone (*n* = 14) or biochemical remission alone (*n* = 17) demonstrated similar rates of UCRC at approximately 30%. Notably, among those achieving both SFCR and biochemical remission (*n* = 14), only 13% experienced UCRC. Interaction terms analysis found that the additional hazard reduction with simultaneous SFCR and biochemical remission was multiplicative, but not formally synergistic (*P*= .718).

**Figure 1:**
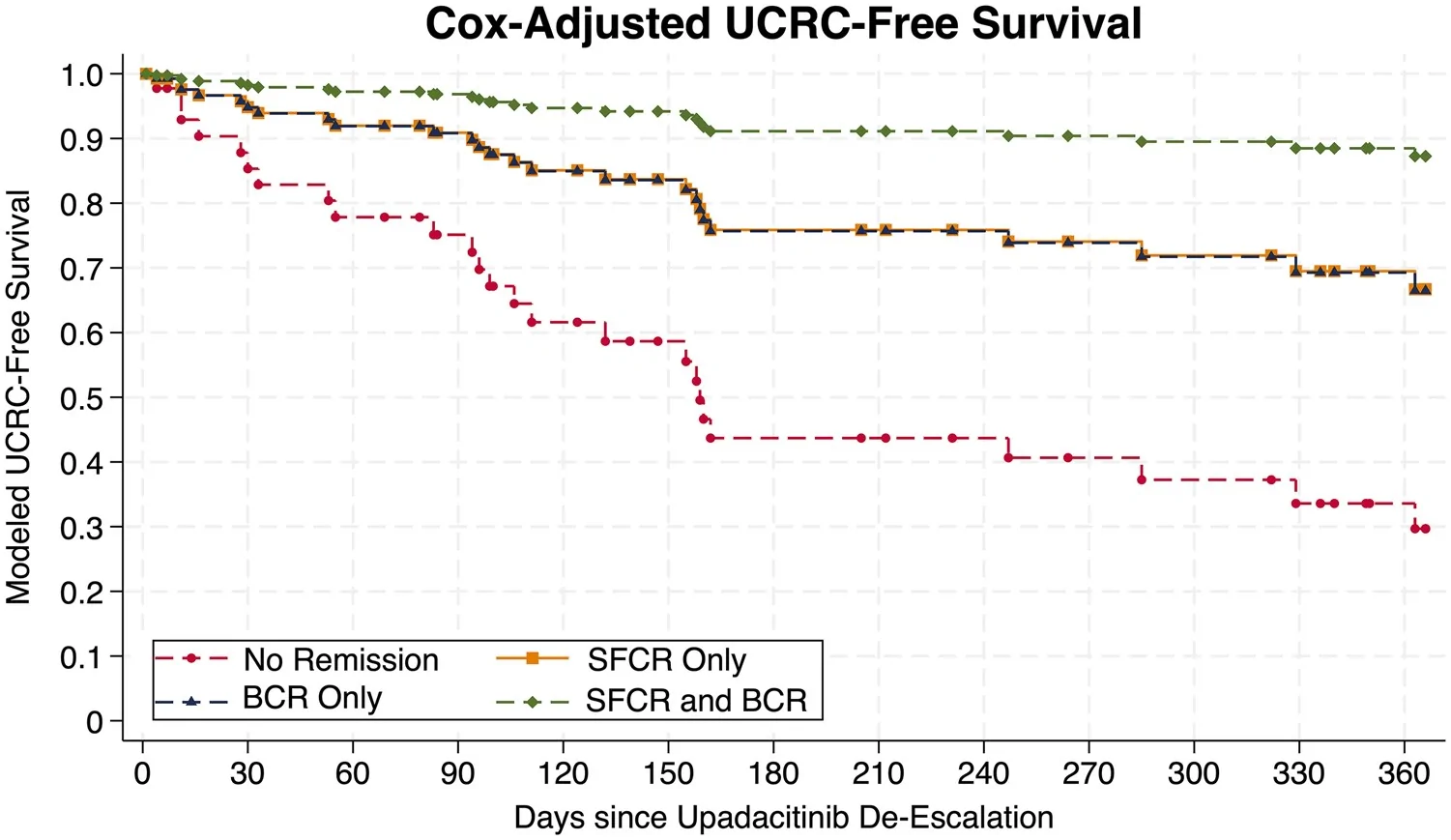
Cox-adjusted survival curves of modeled UCRC-free survival over 1 year of follow-up after upadacitinib de-escalation, with stratification by the presence of SFCR and biochemical remission. The lowest curve (circular markers) represents modeled UCRC-free survival in patients with neither SFCR nor biochemical remission. The 2 curves above this represent modeled UCRC-free survival among patients with SFCR only (square markers) and biochemical remission only (triangular markers). The uppermost curve (diamond markers) represents modeled UCRC-free survival among patients demonstrating both SFCR and biochemical remission.

ROC curves were created to assess the sensitivity and specificity of the biochemical markers for predicting UCRC (Figure 2, Table 4). J-statistic calculation showed an optimal cutpoint for CRP of 12.7 mg/L (AUC = 0.69, sensitivity 40%, specificity 93%) and FC of 58.7 μg/g (AUC = 0.71, sensitivity 100%, specificity 47%). A FC cutoff >150 μg/g resulted in a sensitivity of 71% and a specificity of 65%.

**Figure 2:**
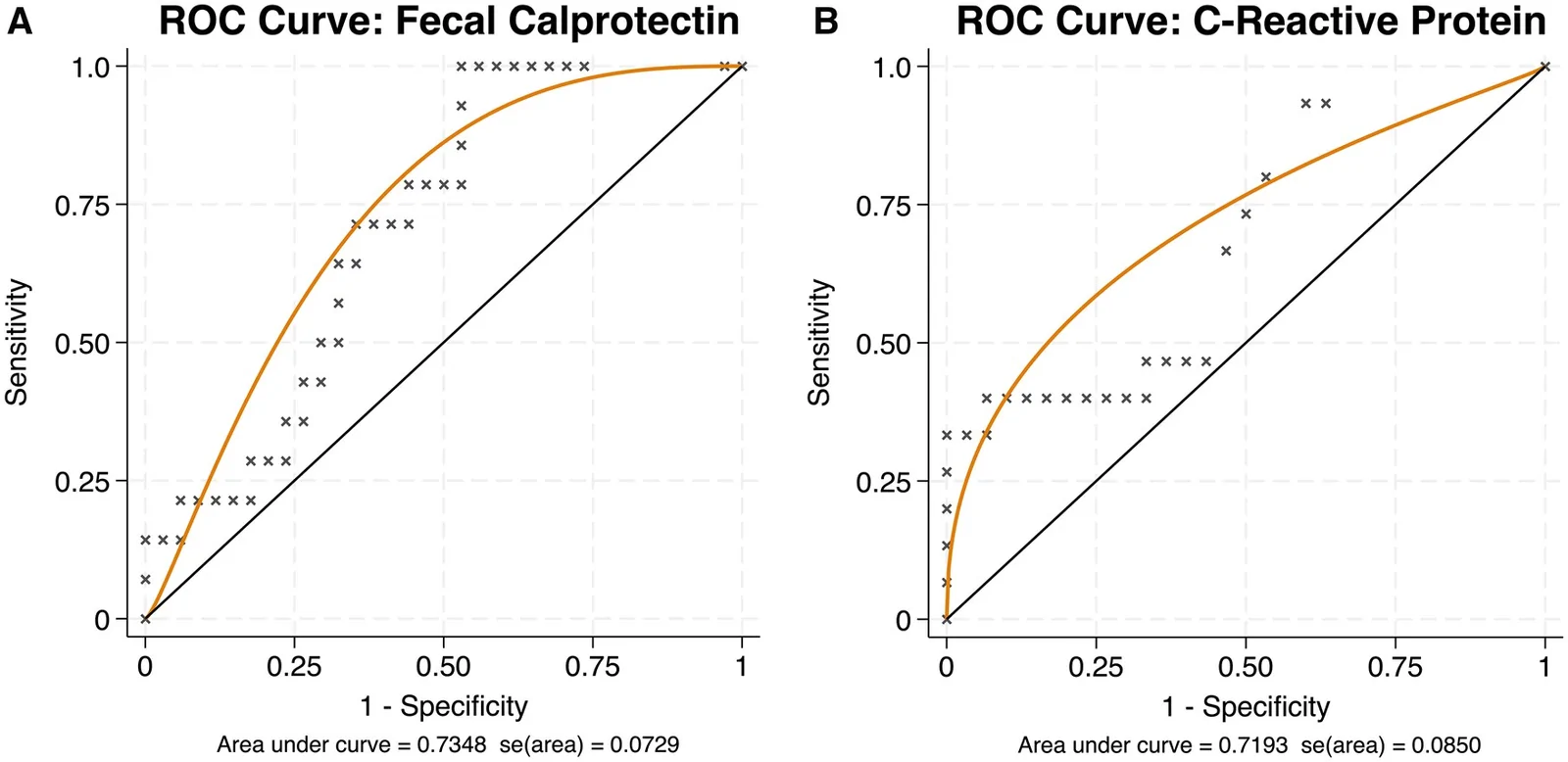
Receiver operating characteristic (ROC) curves showing the sensitivity and specificity of various values of CRP and FC, with empirically observed values indicated by x-shaped markers. The orange curve on each plot represents a parametric curve of best fit calculated via a binormal model.

**Table 4:** Calculated cutoffs of FC and CRP for UCRC prediction.

Biochemical marker	Cutpoint	J-Statistic	AUC	Sensitivity	Specificity
**Fecal calprotectin**	58.7 μg/g	1.47	0.71	1.00	0.47
**C-reactive protein**	12.7 mg/L	1.33	0.69	0.40	0.93

Youden’s J-statistic calculations to determine optimal cutpoints for maximizing predictive value of FC and CRP for UCRC.

### Safety and upadacitinib-related adverse events

Among the 70 patients who completed upadacitinib de-escalation, 17 patients (24.3%) had one or more documented drug-related adverse events during the observation period. Adverse events were predominantly mild, including acne (*n* = 10), weight gain (*n* = 5), non-zoster infections (*n* = 3; 2 COVID-19 infections, 1 fungal vaginitis), arthralgias (*n* = 1), and nonspecific abdominal pain (*n* = 1). No MACE, VTE, herpes zoster reactivation, gastrointestinal perforations, or severe bleeding events occurred during the observation period.

Adverse events occurred in 19.4% (7/36) of patients receiving standard 8-week induction, 25.0% (4/16) receiving 9-16 weeks, and 33.3% (6/18) receiving extended induction beyond 16 weeks (Table S1). On univariable analysis, duration of upadacitinib induction was not significantly associated with therapy-related adverse events (*P*=.539).

## Discussion

In this retrospective, real-world cohort study, we demonstrated that achieving SFCR and biochemical remission prior to upadacitinib dose de-escalation from 45 mg daily independently predicted a lower risk of UCRC, whereas induction duration was not significantly associated with UCRC risk. These findings suggest that the timing of upadacitinib dose de-escalation should be guided by clinical and biochemical targets, rather than a predetermined duration of induction as is recommended on the FDA label.^11^

In our cohort, 35.7% of patients experienced a UCRC following de-escalation, highlighting the need for strategies to optimize clinical outcomes for UC patients treated with upadacitinib, a drug often employed for patients with prior advanced therapy exposures or a high risk for hospitalization and colectomy.^6^^,^^11^ Nonetheless, despite higher biochemical activity (FC) at week 4-8 in patients whose induction duration extended beyond 8 weeks, the proportion of patients experiencing UCRC was not significantly different across induction durations. These findings highlight that patients requiring extended induction for persistent disease activity can ultimately achieve long-term de-escalation success rates similar to standard 8-week responders.

Prior work from the randomized RIVETING trial as well as retrospective data demonstrated that the presence of any active endoscopic disease was associated with a higher likelihood of disease relapse following tofacitinib dose de-escalation among patients in stable remission in a maintenance phase.^17^^,^^24^ While endoscopic assessment remains a gold standard for evaluating mucosal inflammation, performing an endoscopy prior to de-escalation of upadacitinib for every patient utilizing the therapy for induction of remission may be clinically impractical and cost prohibitive. Our study addresses this gap by demonstrating that noninvasive clinical assessments, such as the PRO2 score and biochemical markers (CRP and FC), offer a feasible alternative to endoscopy for identifying patients at risk of UCRC following upadacitinib de-escalation. Furthermore, we found that the simultaneous achievement of both SFCR and biochemical remission prior to de-escalation may confer an additional hazard reduction in a multiplicative manner compared to either metric alone.

Interestingly, baseline endoscopic disease severity, disease distribution, and a history of exposure to other advanced therapies were not associated with an increased risk of UCRC following upadacitinib de-escalation. Our findings mirror registration data, where maintenance of clinical remission after dose de-escalation was largely consistent regardless of baseline disease activity or prior TNFi failure.^15^ Notably, those trials also demonstrated that upadacitinib induction remains highly effective across these difficult-to-treat subgroups.^10^ Our results extend this observation, suggesting that a patient’s current inflammatory state should be the primary guide for upadacitinib dosing de-escalation.

Using ROC analysis, we found a threshold of FC below 58.7 µg/g (sensitivity 100%, specificity 47%) that identified patients with exceptionally low risk of disease relapse and a threshold of CRP above 12.7 mg/L (sensitivity 40%, specificity 93%) at which UCRC were more likely when upadacitinib dose de-escalation was undertaken. The negative predictive value of FC in this setting deserves particular attention. Current clinical practice guidelines utilize a reference FC threshold under 150 µg/g as defining biochemical remission in UC.^25^ A meta-analysis of 25 studies demonstrated substantial variability in optimal cutoff values depending on the clinical context, with lower thresholds often providing greater sensitivity for detecting residual mucosal inflammation.^26^ In this study, UCRC risk increased at FC values well below the 150 µg/g benchmark, suggesting that stricter biochemical remission targets may minimize risk of relapse after upadacitinib dose de-escalation.

Based on these findings, clinicians could consider protocolizing clinical and biochemical reassessment of UC disease activity prior to completion of 8 weeks of upadacitinib induction. Depending on local practice conditions, week 6 may be a pragmatic time point for this assessment, as it provides a buffer to account for laboratory turnaround time and potential pharmacy reauthorization barriers without causing treatment interruption. Furthermore, while real-world patient adherence to FC testing remains low compared to routine serum monitoring,^27^ combining clinical evaluation with serum biomarkers (CRP) could offer a practical alternative to guide the decision for upadacitinib de-escalation.

We also observed an association between white race and improved UCRC-free survival. This finding may signify an outcome disparity but should be interpreted cautiously due to the demographic composition and size of our cohort. It also highlights the need for validation in more diverse populations to determine if potential disparities influence upadacitinib maintenance outcomes.

Several limitations should be acknowledged. The study’s retrospective and single-center design may limit generalizability. Variability in FC assays, particularly when testing was performed at external laboratories, may have introduced measurement heterogeneity, although this reflects routine clinical practice. In addition, most patients de-escalated to a maintenance dose of 30 mg rather than 15 mg, suggesting that the identified biomarker thresholds may not be applicable to alternative prescribing patterns. Furthermore, because our analysis was restricted to patients who completed at least 8 weeks of induction therapy, we could not evaluate whether de-escalation prior to 8 weeks is safe or effective in patients who achieve early SFCR and biochemical remission. Finally, while both SFCR and biochemical remission demonstrated strong prognostic value in this cohort, these data do not address the question of whether acting on these findings would alter rates of UCRC. Prospective studies are therefore needed to determine whether a clinical or biomarker-guided de-escalation strategy, such as delaying dose de-escalation until capturing SFCR or biochemical remission, reduces UCRC compared with standard care.

## Conclusion

SFCR and biochemical remission prior to dose de-escalation of upadacitinib from 45 mg daily predicted lower risk of UCRC, while the length of induction dosing, endoscopic disease severity prior to induction, disease distribution, and prior exposure to advanced therapies were not associated with UCRC following de-escalation. Biochemical targets, specifically FC below 58.7 µg/g and CRP below 12.7 mg/L, may help identify patients most likely to avoid UCRC after dose de-escalation. Prospective studies with larger and more diverse cohorts are needed to validate these observations and to refine personalized maintenance dosing of upadacitinib for UC.

## Supplementary Material

otag111_Supplementary_Data

## Data Availability

The data underlying this article cannot be shared publicly due to institutional IRB restrictions. De-identified data will be shared upon reasonable request to the corresponding author subject to institutional approval.

## References

[1] Virtanen AT , Haikarainen T, Raivola J, Silvennoinen O. Selective JAKinibs: prospects in inflammatory and autoimmune diseases. BioDrugs. 2019;33:15-32. 10.1007/s40259-019-00333-w PMC6373396 30701418

[2] Sandborn WJ , Ghosh S, Panes J, et al Study A3921063 Investigators. Tofacitinib, an oral Janus kinase inhibitor, in active ulcerative colitis. N Engl J Med. 2012;367:616-624. 10.1056/NEJMoa1112168 22894574

[3] Sandborn WJ , Su C, Sands BE, et al OCTAVE Induction 1, OCTAVE Induction 2, and OCTAVE Sustain Investigators. Tofacitinib as induction and maintenance therapy for ulcerative colitis. N Engl J Med. 2017;376:1723-1736. 10.1056/NEJMoa1606910 28467869

[4] Baumgart DC , Berre CL. Newer biologic and small-molecule therapies for inflammatory bowel disease. N Engl J Med. 2021;385:1302-1315. 10.1056/NEJMra1907607 34587387

[5] Loftus EV , Panés J, Lacerda AP, et al Upadacitinib induction and maintenance therapy for Crohn’s disease. N Engl J Med. 2023;388:1966-1980. 10.1056/NEJMoa2212728 37224198

[6] Rubin DT , Ananthakrishnan AN, Siegel CA, Barnes EL, Long MD. ACG clinical guideline update: ulcerative colitis in adults. Am J Gastroenterol. 2025;120:1187-1224. 10.14309/ajg.0000000000003463 40701556

[7] Voss J , Graff C, Schwartz A, et al THU0127 pharmacodynamics of a novel JAK1 selective inhibitor in rat arthritis and anemia models and in healthy human subjects. Ann Rheum Dis. 2014;73:222. 10.1136/annrheumdis-2014-eular.3823

[8] Parmentier JM , Voss J, Graff C, et al In vitro and in vivo characterization of the JAK1 selectivity of upadacitinib (ABT-494). BMC Rheumatol. 2018;2:23. 10.1186/s41927-018-0031-x PMC6390583 30886973

[9] Sandborn WJ , Ghosh S, Panes J, et al Efficacy of upadacitinib in a randomized trial of patients with active ulcerative colitis. Gastroenterology. 2020;158:2139-2149. 10.1053/j.gastro.2020.02.030 32092309

[10] Danese S , Vermeire S, Zhou W, et al Upadacitinib as induction and maintenance therapy for moderately to severely active ulcerative colitis: results from three phase 3, multicentre, double-blind, randomised trials. Lancet. 2022;399:2113-2128. 10.1016/s0140-6736(22)00581-5 35644166

[11] Label: RINVOQ- Upadacitinib Tablet, Extended Release. U.S. Food and Drug Administration. 2026. Accessed October 13, 2026. https://dailymed.nlm.nih.gov/dailymed/drugInfo.cfm?setid=2966aec7-2ef0-923c-d8ff-fe1a957bf095

[12] Panaccione R , Danese S, Zhou W, et al Efficacy and safety of upadacitinib for 16-week extended induction and 52-week maintenance therapy in patients with moderately to severely active ulcerative colitis. Aliment Pharmacol Ther. 2024;59:393-408. 10.1111/apt.17816 38010661

[13] García MJ , Brenes Y, Vicuña M, et al Persistence, effectiveness, and safety of upadacitinib in crohn’s disease and ulcerative colitis in real life: Results from a Spanish nationwide study (ureal study). Am J Gastroenterol. 2024;120:1593-1604. 10.14309/ajg.0000000000003243 39588977

[14] Ho AHY , Choi D, Mathew AJ, et al Real-world experience of upadacitinib reinduction and high-dose maintenance therapy in inflammatory bowel disease. Clin Gastroenterol Hepatol. 2026;24:763-771. 10.1016/j.cgh.2025.07.042 40782945

[15] Vermeire S , Danese S, Zhou W, et al Efficacy and safety of upadacitinib maintenance therapy for moderately to severely active ulcerative colitis in patients responding to 8 week induction therapy (U-ACHIEVE maintenance): overall results from the randomised, placebo-controlled, double-blind, phase 3 maintenance study. Lancet Gastroenterol Hepatol. 2023;8:976-989. 10.1016/S2468-1253(23)00208-X 37683686

[16] Kandasamy C , Rotondo-Trivette S, Cordova N, et al Tu1838: real-world outcomes of extended induction and reinduction with upadacitinib for treatment of ulcerative colitis. Gastroenterology. 2025;169:S-1516. 10.1016/S0016-5085(25)04367-7

[17] Vermeire S , Su C, Lawendy N, et al Outcomes of tofacitinib dose reduction in patients with ulcerative colitis in stable remission from the randomised RIVETING trial. J Crohns Colitis. 2021;15:1130-1141. 10.1093/ecco-jcc/jjaa249 PMC8256630 33290538

[18] Pauwels RWM , van der Woude CJ, Nieboer D, et al CEASE Study Group. Prediction of relapse after anti-tumor necrosis factor cessation in Crohn’s disease: individual participant data meta-analysis of 1317 patients from 14 studies. Clin Gastroenterol Hepatol. 2022;20:1671-1686. 10.1016/j.cgh.2021.03.037 33933376

[19] Buisson A , Mak WY, Andersen MJ, et al Faecal calprotectin is a very reliable tool to predict and monitor the risk of relapse after therapeutic de-escalation in patients with inflammatory bowel diseases. J Crohns Colitis. 2019;13:1012-1024. 10.1093/ecco-jcc/jjz023 PMC6939876 30726887

[20] Molander P , Färkkilä M, Ristimäki A, et al Does fecal calprotectin predict short-term relapse after stopping TNFα-blocking agents in inflammatory bowel disease patients in deep remission? J Crohns Colitis. 2015;9:33-40. 10.1016/j.crohns.2014.06.012 25052347

[21] Yin J , El-Najjar Y, Cordova N, et al Short-term use of upadacitinib in combination with biologic therapy for inducing clinical remission in patients with active inflammatory bowel disease. Inflamm Bowel Dis. 2024;30:1914-1916. 10.1093/ibd/izae105 PMC12102465 38767984

[22] Jairath V , Khanna R, Zou GY, et al Development of interim patient-reported outcome measures for the assessment of ulcerative colitis disease activity in clinical trials. Aliment Pharmacol Ther. 2015;42:1200-1210. 10.1111/apt.13408 26388424

[23] Yu A , Ha NB, Shi B, Cheng YW, Mahadevan U, Beck KR. Real-world experience with tofacitinib dose de-escalation in patients with moderate and severe ulcerative colitis. Clin Gastroenterol Hepatol. 2023;21:3115-3124. 10.1016/j.cgh.2023.05.001 37187323

[24] Singh S , Ananthakrishnan AN, Nguyen NH, et al AGA Clinical Guidelines Committee. AGA clinical practice guideline on the role of biomarkers for the management of ulcerative colitis. Gastroenterology. 2023;164:344-372. 10.1053/j.gastro.2022.12.007 36822736

[25] Shi J-T , Chen N, Xu J, et al Diagnostic accuracy of Fecal Calprotectin for predicting relapse in Inflammatory Bowel Disease: a Meta-Analysis. JCM. 2023;12:1206. 10.3390/jcm12031206 PMC9917450 36769850

[26] Khakoo NS , Lewis A, Roldan GA, et al Patient adherence to fecal calprotectin testing is low compared to other commonly ordered tests in patients with inflammatory bowel disease. Crohns Colitis 360. 2021;3:otab028. 10.1093/crocol/otab028 PMC9802323 36776647

