# Supplementary material for: Real-world success of de-escalating upadacitinib induction dosing in ulcerative colitis is predicted by steroid-free clinical and biochemical remission, not induction duration
Source: Crohns Colitis 360. 2026 Oct 29;8(4):otag111. doi: 10.1093/crocol/otag111 (PMC13730274; doi:10.1093/crocol/otag111)
Supplement: Supplementary file 1 [file otag111_supplementary_data.pdf]

	Total	8 Weeks	9-16 Weeks	>16 Weeks	p
**TOTAL**	70	36 (51.4%)	16 (22.9%)	18 (25.7%)	-
**Male**					
Male	38 (54.3%)	17 (47.2%)	8 (50.0%)	13 (72.2%)	0.204
Female	32 (45.7%)	19 (52.8%)	8 (50.0%)	5 (27.8%)	
**Race**					
White	56 (80.0%)	29 (80.6%)	12 (75.0%)	15 (83.3%)	0.772
Black	6 (8.6%)	2 (5.6%)	2 (12.5%)	2 (11.1%)	
Other/Unknown	8 (11.4%)	5 (13.9%)	2 (12.5%)	1 (5.6%)	
**Hispanic**	12 (17.1%)	7 (19.4%)	2 (12.5%)	3 (16.7%)	0.916
**Body Mass Index (kg/m^2)**	26.3 [22.7, 30.1]	25.3 [22.6, 30.0]	30.0 [25.7, 31.9]	25.3 [23.0, 27.8]	0.142
**Age at Induction (years)**	37.5 [31, 49]	40.5 [31.5, 52]	41 [28, 50]	35 [32, 45]	0.749
**Diagnosis Duration (years)**	9 [5, 16]	11 [6, 15.5]	9 [7, 19]	8 [4, 12]	0.391
**Disease Distribution**					
Proctitis	1 (1.4%)	0 (0.0%)	1 (6.3%)	0 (0.0%)	0.380
Left-Sided	14 (20.0%)	7 (19.4%)	2 (12.5%)	5 (27.8%)	
Extensive	55 (78.6%)	29 (80.6%)	13 (81.3%)	13 (72.2%)	
**Prior Advanced Therapies**	3 [2, 4]	3 [2, 4]	3 [2.5, 4.5]	3 [2, 4]	0.594
**Prior Infliximab Exposure**	49 (70.0%)	25 (69.4%)	10 (62.5%)	14 (77.8%)	0.621
**Hospitalized at Induction**	10 (14.3%)	5 (13.9%)	2 (12.5%)	3 (16.7%)	1.000
**Systemic Steroids at Induction**	28 (40.0%)	14 (38.9%)	8 (50.0%)	6 (33.3%)	0.601
**Dual Advanced Therapy at Induction**	10 (14.3%)	6 (16.7%)	1 (6.3%)	3 (16.7%)	0.668
**Pre-Induction Clinical Disease Activity**					
Remission	16 (22.9%)	9 (25.0%)	4 (25.0%)	3 (16.7%)	0.822
Mild to Moderate	2 (2.9%)	2 (5.6%)	0 (0.0%)	0	
Moderate to Severe	52 (74.3%)	25 (69.4%)	12 (75.0%)	15 (83.3%)	
**Pre-Induction CRP (mg/L)**	10.4 [3.0, 29.5]	10.6 [4.1, 34.5]	14.8 [4.0, 32.1]	6.0 [1.1, 21.0]	0.430
**Pre-Induction FC (ug/g)**	978.5 [416.8, 2300.5]	972.9 [173.0, 2355.9]	972.7 [525.0, 3991.8]	1070.0 [493.0, 1966.5]	0.824
**Pre-Induction Biochemical Remission**	8 (11.4%)	6 (16.7%)	2 (12.5%)	0 (0.0%)	0.202
**Pre-Induction Mayo Endoscopic Score**					
Mayo 0-1	4 (5.7%)	3 (8.3%)	1 (6.3%)	0 (0.0%)	**0.016**
Mayo 2	17 (24.3%)	7 (19.4%)	7 (43.8%)	3 (16.7%)	
Mayo 3	25 (35.7%)	14 (38.9%)	1 (6.3%)	10 (55.6%)	
**Week 4-8 Clinical Disease Activity**					
Remission	23 (32.9%)	12 (33.3%)	5 (31.3%)	6 (33.3%)	0.774
Mild to Moderate	5 (7.1%	4 (11.1%)	1 (6.3%)	0 (0.0%)	
Moderate to Severe	42 (60.0%)	20 (55.6%)	10 (62.5%)	12 (66.7%)	
**Week 4-8 CRP (mg/L)**	1.5 [0.5, 9.0]	0.8 [0.2, 4.0]	2.6 [0.6, 15.5]	7.6 [1.2, 26.5]	0.123
**Week 4-8 FC (ug/g)**	143.8 [37.0, 470.0]	74.8 [15.0, 160.0]	233.5 [89.6, 556.0]	475.0 [321.0, 2150.0]	**0.007**
**Upadacitinib-Related Adverse Event**	17 (24.3%)	7 (19.4%)	4 (25.0%)	6 (33.3%)	0.539

Supplemental Table 1: Clinical and demographic data for patients has been stratified by induction duration. Results are reported as either number (percent) or median [IQR] for categorical and continuous variables, respectively. P-values are calculated via Chi-square or Kruskal-Wallis tests for categorical variables and Wilcoxon rank-sum tests for continuous variables, comparing clinical and demographic characteristics by induction duration.

Candidate Variable	Hazard Ratio	95% CI	Univariable p
Male (vs. Female)	1.871	[0.807, 1.608]	0.275
White (vs. Non-White) Race	0.331	[0.142, 0.771]	**0.010**
Hispanic	0.699	[0.208, 2.345]	0.562
BMI *(continuous)*	1.021	[0.955, 1.091]	0.548
Extensive Disease (vs. Other)	1.691	[0.580, 4.932]	0.336
Age at Induction *(continuous)*	0.975	[0.947, 1.005]	0.103
Age at Diagnosis *(continuous)*	0.977	[0.948, 1.008]	0.146
Years Since Diagnosis *(continuous)*	0.996	[0.943, 1.053]	0.899
Prior Advanced Therapy Count			
No Prior AT	Reference	Reference	Reference
1 Prior AT	1.392	[0.359, 5.393]	0.632
2 Prior AT	1.053	[0.305, 3.642]	0.935
Hospitalized During Induction	0.793	[0.234, 2.622]	0.692
Systemic Steroids at Induction	1.074	[0.487, 2.369]	0.860
Dual Therapy at Induction	0.359	[0.085, 1.527]	0.166
Pre-Induction RBS *(continuous)*	1.221	[0.772, 1.929]	0.394
Pre-Induction SFS *(continuous)*	1.053	[0.733, 1.515]	0.779
Pre-Induction CRP *(continuous)*	1.077	[0.997, 1.017]	0.148
Pre-Induction FC *(continuous)*	1.000	[1.000, 1.000]	0.390
Pre-Induction Mayo Subscore			
Mayo 0-1	Reference	Reference	Reference
Mayo 2	0.638	[0.077, 5.318]	0.678
Mayo 3	0.387	[0.046, 3.227]	0.380
Induction Dosing Duration			
8 Weeks	Reference	Reference	Reference
9-16 Weeks	1.717	[0.710, 4.155]	0.230
>16 Weeks	0.655	[0.213, 2.011]	0.460
Steroid-Free Clinical Remission at De-Escalation	0.447	[0.186, 1.072]	**0.071**
Biochemical Remission at De-Escalation	0.453	[0.189, 1.087]	**0.076**

Supplemental Table 2: Univariable Cox proportional hazard modeling showing unadjusted hazard ratios by clinical and demographic variables. Variables were selected for inclusion in multivariable analysis based on having $p < 0.1$.
